# Recent Advances in Gene Therapy for Cardiac Tissue Regeneration

**DOI:** 10.3390/ijms22179206

**Published:** 2021-08-26

**Authors:** Yevgeniy Kim, Zharylkasyn Zharkinbekov, Madina Sarsenova, Gaziza Yeltay, Arman Saparov

**Affiliations:** Department of Medicine, School of Medicine, Nazarbayev University, Nur-Sultan 010000, Kazakhstan; yevgeniy.kim@nu.edu.kz (Y.K.); zharylkasyn.zharkinbekov@nu.edu.kz (Z.Z.); madina.sarsenova@nu.edu.kz (M.S.); gaziza.yeltay@nu.edu.kz (G.Y.)

**Keywords:** gene therapy, cardiovascular diseases, cardiac regeneration, cardiac reprogramming, therapeutic angiogenesis, growth factors, reactive oxygen species, apoptosis

## Abstract

Cardiovascular diseases (CVDs) are responsible for enormous socio-economic impact and the highest mortality globally. The standard of care for CVDs, which includes medications and surgical interventions, in most cases, can delay but not prevent the progression of disease. Gene therapy has been considered as a potential therapy to improve the outcomes of CVDs as it targets the molecular mechanisms implicated in heart failure. Cardiac reprogramming, therapeutic angiogenesis using growth factors, antioxidant, and anti-apoptotic therapies are the modalities of cardiac gene therapy that have led to promising results in preclinical studies. Despite the benefits observed in animal studies, the attempts to translate them to humans have been inconsistent so far. Low concentration of the gene product at the target site, incomplete understanding of the molecular pathways of the disease, selected gene delivery method, difference between animal models and humans among others are probable causes of the inconsistent results in clinics. In this review, we discuss the most recent applications of the aforementioned gene therapy strategies to improve cardiac tissue regeneration in preclinical and clinical studies as well as the challenges associated with them. In addition, we consider ongoing gene therapy clinical trials focused on cardiac regeneration in CVDs.

## 1. Introduction

Cardiovascular diseases (CVDs) remain the principal cause of mortality and morbidity worldwide. In 2019 alone, it was estimated that there were approximately 18.6 million deaths due to CVDs, which accounted for almost one third of all global mortalities [1]. At the same time, the prevalence of CVDs exceeded half a billion cases in 2019 [1]. A more concerning fact about morbidity and mortality related to CVDs is that they are continuing to grow. According to the Global Burden of Disease 2019 Study, the number of deaths caused by CVDs increased by approximately 6.5 million for the period between 1990–2019, while the amount of prevalent cases of CVDs almost doubled for the same period [1]. In addition to the huge impact on people’s health and quality of life, CVDs are blameworthy for huge economic losses. In the countries of the European Union in 2017, total spending associated with CVDs were estimated to be €210 billion euro [2]. In the USA in 2017, CVD-related healthcare and productivity loss costs exceeded $320 billion in 2017 [3]. The estimated healthcare costs are predicted to increase more than 2.5-fold, reaching $818 billion, by 2030 [3].

The standard treatments for CVDs include pharmacologic agents, therapeutic devices and surgical interventions. Although these therapies improve survival, reduce symptoms, and improve quality of life, they do not reverse the pathologic processes associated with CVDs and because of this, the health condition of most patients deteriorate with time [4]. Advances in cellular and molecular biology shed light on the pathogenetic and pathophysiologic mechanisms of CVDs and give promising opportunities for the use of novel therapies to combat heart and vascular diseases. Since these therapies are targeted towards the molecular cause of a specific CVD, they could hold a promising approach to cure the disease. Such treatments include cell-based therapies, therapies with growth factors and other bioactive molecules, biomaterials, and others [5,6,7,8]. Another therapeutic strategy that could likely improve the outcome of CVDs is gene therapy.

Gene therapy has been investigated for the treatment of a multitude of CVDs and CVD-related conditions such as atherosclerosis, coronary artery disease (CAD), myocardial infarction (MI), peripheral arterial disease (PAD), hypertension, various types of arrhythmias, heart failure (HF), restenosis of coronary stents, failure of vein grafts, and others [9,10,11,12,13]. Preclinical trials involving gene therapy for the aforementioned conditions were largely successful, however, there are a number of challenges associated with the translation of this therapeutic modality to clinical use [9]. Although there were several clinical trials for gene therapy, which showed positive results in terms of safety and certain therapeutic efficiency (specifically, trials for CAD, PAD, and HF), many other randomized-controlled studies failed to show the benefits of gene therapy over standard treatments [9]. This lack of consistent results in clinical trials can be attributed to multiple reasons including low concentration of the transferred gene or its product at the target site, incomplete knowledge of the disease mechanism and hence wrong gene therapy strategy, significant differences between animal models and human subjects, and others [4,9].

In this review, we discuss the latest preclinical findings and clinical trials related to the application of gene therapy for the treatment of CVDs. Specifically, we consider how cardiac gene therapy can be used to enhance angiogenesis, remodel scar tissue, alleviate production of reactive oxygen species, and prevent apoptosis.

## 2. Cardiac Tissue Regeneration in Adult Heart

### 2.1. Mechanisms of Cardiac Tissue Regeneration

The myocardium of adult mammals including humans has an extremely low regenerative capacity due to low proliferation rates of cardiomyocytes and scarcity of cardiac stem cells. In fact, for a long time, it was believed that cardiomyocytes in the adult heart are completely unable to proliferate. However, several studies using carbon-14 dating have detected a slow but persistent turnover of cardiomyocytes throughout life [14,15,16]. According to the American Heart Association’s 2017 Consensus Statement, the annual turnover rate was reported to be between 0.5% and 2.0% [17]. Moreover, after injury to the heart muscle, the cardiomyocyte proliferation rate increases several times [18]. In contrast to adults, the neonatal mammalian heart possesses an outstanding capacity for regeneration. In the last decade, there have been multiple studies that demonstrated a robust regenerative response following apical resection and ischemic injury in mice shortly after birth [19,20,21,22,23]. The ability of neonatal myocardium to completely regenerate was also established in porcine and rat models of MI [24,25,26]. Importantly, neonates exhibit this remarkable regenerative capacity only for a brief period of time after birth, i.e., it is rapidly lost two to seven days postnatally [19,25,27].

Cardiac tissue regeneration is a very complex process that involves multiple genes and signaling pathways. Many reports showed that myocardial recovery is mainly mediated by the proliferation of existing cardiomyocytes with little contribution from cardiac progenitor cells [19]. Cardiomyocyte proliferation in turn is regulated by multiple cells and signaling interactions that is demonstrated by recent gene expression and chromatin modification profiling studies [28,29]. Using single-cell RNA sequencing, Wang and colleagues have identified 22 non-cardiomyocyte cell types involved in cardiac regeneration, including endothelial cells, fibroblasts, epicardial cells, pericytes, smooth muscle cells, and a number of immune cells—namely, macrophages, monocytes, dendritic cell-like cells, granulocytes, T cells, B cells, and glial cells [29]. In another recent transcriptome profiling study, two novel regulators of neonatal heart regeneration were found, specifically, C-C motif chemokine ligand 24 (CCL24) and insulin-like growth factor 2 messenger RNA-binding protein 3 (Igf2bp3) [28].

The main players associated with cardiac tissue regeneration are growth factors, cell cycle regulators, and miRNAs. In addition, several intrinsic signaling pathways were found to be essential in cardiomyocyte proliferation and heart regeneration. Neuregulin 1 (NRG1) is probably the most studied growth factor involved in heart development and regeneration [30]. It signals via Erb-B2 receptor tyrosine kinases 2-4 (ERBB2-4) and mediates the formation of a normal heart during embryonic development as well as promotes proliferation of cardiomyocytes after cardiac injuries [31]. Other growth factors reported to have a role in cardiac regeneration are follistatin-like 1 and insulin-like growth factor 2, which can mediate cell cycle re-entry of cardiomyocytes [30]. Another group of molecules that participates in cardiac regeneration are cell cycle regulators, i.e., cyclins and cyclin-dependent kinases (CDKs). Earlier studies found that cyclins A2, D1, and D2 can promote cardiomyocyte proliferation in adult hearts [30]. A recent study by Mohamed and colleagues discovered that in addition to the cell cycle regulators mentioned above, cyclins B1 and D1 as well as CDK 1 and CDK 4 are capable of inducing cell division in adult cardiac tissue [32]. This effect was evidenced by the overexpression studies as well as inhibition of Wee1 and TGFβ, which directly or indirectly suppress the activity of the aforementioned cell cycle regulators. Yet another class of molecules involved in heart regeneration are miRNAs. Recently, a large-scale screen of miRNAs revealed that there are 96 miRNAs that can mediate cardiomyocyte proliferation [33]. The same study discovered that the majority of these miRNAs acted via Hippo pathway and depended on the pathway’s effector YAP. Interestingly, individual silencing of the miRNA did not suppress cell division indicating that none of these miRNAs on its own is essential for cardiomyocyte proliferation. The Hippo pathway is likely the most investigated intrinsic pathway that regulates cardiac tissue regeneration and cardiomyocyte proliferation. This evolutionary conserved pathway controls heart size by inhibiting cardiomyocyte division [30]. In addition, the Hippo pathway was shown to suppress cardiac tissue regeneration since the deletion of some of its components enhanced heart repair. Hippo pathway’s downstream effector YAP, by contrast, was found to have pro-proliferative effects on cardiomyocytes. Thus, Monroe and colleagues recently demonstrated that YAP5SA causes expression of fetal genes and promotes proliferation in mouse cardiomyocytes [34].

The loss of cardiac regenerative capacity shortly after birth is caused by an exit of cardiomyocytes from the cell cycle and an entering into quiescent state. It is suggested that a change in extracellular matrix (ECM) and cytoskeleton architecture is a main factor that is culpable for the transition of cardiomyocytes to a senescent state. In particular, as cardiac cytoskeleton and ECM become stiffer and more organized and stable, it becomes more difficult for cell division to occur [35]. This was confirmed in a recent study by Notari and colleagues [27], in which the transcriptome of mice on the first and second postnatal days were compared. The study revealed that P2 murine neonates could not regenerate myocardium after resection of about 15% of the apex and responded with fibrosis. Importantly, cardiomyocytes of P2 mice did not lose their proliferative capacity, which indicates that the loss of regenerative ability was not associated with quiescence of the cells. On the other hand, a transcriptome analysis found a significant difference in the expression of genes related to ECM and cytoskeleton between P1 and P2 neonates. Specifically, mice on the second postnatal day overexpressed the aforementioned genes [27]. These findings support the theory that ECM and cytoskeleton growth and development are responsible for the loss of cardiac regenerative capacity shortly after birth.

### 2.2. Overview of Strategies to Enhance Cardiac Tissue Regeneration

Given the fact that poor cardiac regeneration lies in the root of the pathogenesis and pathophysiology of many CVDs, multiple therapeutic strategies have been suggested in order to enhance the regenerative capacity of the adult heart. A multitude of approaches that were shown to improve cardiac repair and regeneration can be roughly divided into three main categories, namely, bioactive molecules and secretory factors, cell-based strategies, and biomaterials. Paracrine effects of growth factors and cytokines were found to improve the regeneration of cardiac tissue. For instance, vascular endothelial factor A (VEGF-A) and fibroblast growth factor 2 (FGF2) enhanced cardiac repair by reducing scar size and mediating angiogenesis in animal models of MI [36]. In addition to growth factors and cytokines, microRNAs (miRNAs) have also been considered for regenerative therapy. MiRNAs are highly conserved, short, single-stranded non-coding RNA molecules, which control gene expression at the post-transcriptional level [18]. Since miRNAs are important regulators of cardiomyocyte proliferation, it was proposed that their application could promote cardiac regeneration in the adult heart. Specifically, this could be done by the activation of miRNAs that upregulate cardiomyocyte proliferation such as miR-199a and miR-590, or by suppression of miRNAs that inhibit cardiomyocyte division, namely, miR-195, miR-15a, miR-15b, miR-16, and miR-497 [18,36,37]. However, the wide application of growth factors and other secreted molecules for cardiac regeneration is limited by their short half-life.

Early studies involving cell-based approaches for cardiac regeneration utilized skeletal myocytes, resident cardiac-derived cells, bone marrow-derived cells, and mesenchymal stem cells [36,38,39]. Despite certain success achieved by some research groups, the results of preclinical studies with these cells were either inconsistent or failed to further translate to clinical trials [36]. The next stage in the development of cellular therapy for cardiac repair was the application of human embryonic stem cells (hESCs) and human induced pluripotent stem cells (hiPSCs) [40]. In both cases, multiple animal studies reported positive results, i.e., hESCs and hiPSCs differentiated into cardiomyocytes in vitro, survived after transplantation to the injured heart and significantly enhanced cardiac function [38]. In addition to direct benefits of cardiomyocyte replacement, hESCs and hiPSCs contributed to the regeneration of cardiac tissue via paracrine mechanisms secreting factors that promoted cardiomyocyte proliferation and angiogenesis, and inhibited apoptosis and fibrosis [8]. Despite the promising results of cell-based therapy observed in animals, there are a number of challenges associated with its translation to human studies. Firstly, cell transplantation could induce tumorigenesis and life-threatening arrhythmias [36,41]. In addition, it was found that the efficiency of a cell-based approach is significantly limited because of low survival and poor engraftment of the transplanted cells [38,41]. Rapid cell death following transplantation was related to preparation techniques, harsh environment of the injured heart and immune response [36,38]. Current studies involving cell-based strategies are, therefore, focused on approaches that could address the hurdles mentioned above.

The major approaches utilized to optimize stem cell therapy for cardiac regeneration are preconditioning, genetic manipulations, and use of biomaterials. Stem cell preconditioning using hypoxia, chemical and pharmacologic agents, and bioactive molecules was shown to significantly improve survival of the cells after transplantation as well as enhance their beneficial effects on the heart [8,42,43]. The positive effects of preconditioning on stem cells are related to induction of pro-survival and inhibition of apoptotic genes [42]. For instance, hypoxia activates hypoxia-inducible factor-1α (HIF-1α), which in turn promotes the expression of VEGF and angiotensin, two molecules that stimulate angiogenesis [44,45]. At the same time, it suppresses the expression of apoptosis-related proteins Bcl-2 and Bcl-xL [8]. Biomaterials such as nanoparticles, hydrogels, cryogels, coacervate, and scaffolds were also utilized to deliver various cytokines and growth factors for improving tissue regeneration [46,47,48,49] and to enhance the efficiency of cell therapy for cardiac diseases [50]. In multiple preclinical studies on MI, the use of biomaterials combined with stem cells was associated with enhanced survival and engraftment of the cell [7]. Moreover, stem cells combined with biomaterials stimulated cardiomyogenesis and angiogenesis as well as the release of factors that are important for cardiomyocyte survival and proliferation [7,51]. Another strategy to improve cellular therapy for cardiac regeneration is genetic manipulation, which will be further discussed in the upcoming sections of the paper.

Overall, adult cardiac tissue in humans has an extremely low ability to regenerate and because of this, injury to myocardium results in the formation of non-contractile fibrotic tissue eventually leading to higher morbidity and mortality. Cardiac regeneration strategies involving bioactive molecules, cells, and biomaterials attempt to improve the regenerative capacity of adult myocardium.

## 3. Gene Therapy for Cardiac Tissue Regeneration

### 3.1. Gene Therapy with Growth Factors for Improving Angiogenesis

**Figure 1:**
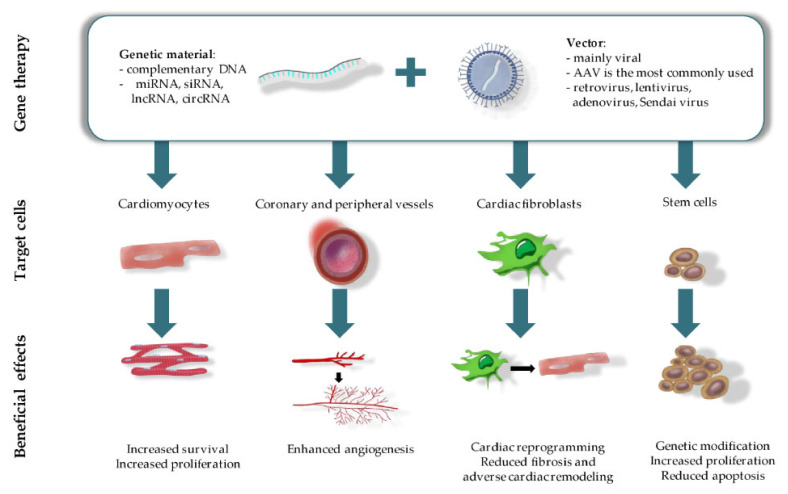
Gene therapy and therapeutic outcomes in CVDs treatment.

Therapeutic angiogenesis can be a potential treatment option for improving cardiac tissue functioning by stimulating blood vessel growth, increasing tissue perfusion and recovery [52]. In this regard, gene-based therapy with a qualitative gene delivery system (a plasmid or viral origin) in combination with adequate pro-angiogenic genes can serve as a promising tool for successful cardiac tissue regeneration (Figure 1). A well-developed gene-based system must include the following requirements: sustained long-term therapeutic effect, ability to target specific cell types and a decreased risk of systemic side-effects as compared to regular pharmacotherapy. To date, gene transfer systems include two approaches such as direct tissue injection and intravascular infusion with or without surgical or catheter-mediated interventions [53]. In addition, for gene therapy in heart diseases, the goal in most cases is to deliver genetic material directly to the myocardium. Expression of the targeted gene needs to be sustained over an extended period of time unless therapy is meant to repair a specific structural defect. Most importantly, the gene must encode a molecule that plays a critical role in the disease pathogenesis such that by altering expression of that gene alone, cardiac function will be improved sufficiently or will favorably alter the disease flow [54]. Thus, gene therapy using appropriate delivery systems, which meet the aforementioned requirements, and pro-angiogenic factors that act in processes such as blood flow, metabolic activity, and cardiac functioning are being applied for cardiac angiogenesis [55]. Among these factors are VEGF, FGF, and hepatocyte growth factor (HGF). In this section, we will focus on the insights from recent growth factor gene-based therapy approaches.

Among the pro-angiogenic factors, VEGF has been the most extensively studied. It is a 45-kD homodimeric glycoprotein that has four main isoforms: VEGF-A (possesses the ability to bind heparin), -B, -C, and -D. There are additional isoforms in VEGF-A: VEGF121, VEGF165, which is the most biologically active, VEGF189 and VEGF206 [56]. Many other known angiogenic factors act at least partly via VEGF-A. VEGF-B has some exceptional features compared to other VEGFs since it induces myocardium-specific angiogenesis without the risk of hyperpermeability and edema. Interestingly, it also seems to alter myocardial energy metabolism and fatty acid uptake and promote cell survival and compensatory hypertrophy [57]. The receptors for VEGF are FLT-1 and FLK-1, which activate intracellular tyrosine kinase. Neuropilin 1 (NP-1) is another receptor for VEGF that binds VEGF165. NP-1 and FLK-1 are key mediators of the phosphoinositide-3-kinase, and Akt (PI3K/Akt) and mitogen-activated protein kinase (MAPK) kinase pathways [58].

VEGF-A isoform has been well studied in a number of preclinical studies and several clinical trials. However, VEGF-B and VEGF-D isoforms have attracted much attention from scientists in the past few years [59]. In this regard, Nurro and colleagues demonstrated an angiogenic capacity of new members of the VEGF family, VEGF-B186 and VEGF-DΔNΔC, in porcine myocardium [60]. Both adenovirus-mediated VEGF-B186 (AdVEGF-B186) and AdVEGF-DΔNΔC gene transfers induced efficient angiogenesis in the myocardium, possibly due to their high solubility in tissues since they do not bind effectively to heparan sulfates, suggesting that they could be suitable candidates for the induction of therapeutic angiogenesis for the treatment of refractory angina. In a study by Huusko and colleagues, it was demonstrated that VEGF-C and VEGF-D are associated with the compensatory ventricular hypertrophy and that adeno-associated virus subtype 9 (AAV9)-VEGF-B186 gene transfer can rescue the function of the failing heart and postpone the transition towards HF in mice [61]. Another strategy of gene therapy is based on the concept of a combined approach using a simultaneous delivery of VEGF165 and HGF genes to alleviate the symptoms of MI in rats [62]. Combined application of these genes resulted in an increase of the number of cardiac stem cells in the peri-infarct area and sporadic proliferation of mature cardiomyocytes. These effects may be explained by the activation of VEGFRs and c-Met (HGF receptor) that initiate mitogenic signaling cascades. Furthermore, it is reported that VEGF-D stimulates both angiogenesis and lymphangiogenesis, which has been also tested in animal models and confirmed by a phase I/IIa clinical study with one-year follow-up [63]. Another clinical study investigated a novel VEGF-D, a new member of the VEGF family called VEGF-DΔNΔC, in 30 patients with refractory angina. This phase I/IIa randomized clinical trial used increasing doses of endocardial adenoviral (ad)-injections with electroanatomical targeting of injections using a NOGA catheter system. AdVEGF-DΔNΔC gene therapy is considered to be safe and well tolerated, and confirmed by a consistent report that lipoprotein (a) level in 50% of patients with refractory angina was significantly decreased [64].

FGF is another angiogenic factor that has been studied in CVDs. FGF is related to a family of 22 identified members of pleiotropic proteins in human and mouse and regulates crucial functions in the heart, ranging from development to homeostasis and disease, and is considered a cardiomyokine [65,66]. The mechanism of action of FGF proteins is based on FGF/FGFR signaling, which plays an important role in angiogenesis and lymphangiogenesis. FGF signaling can influence the whole process of angiogenesis and cardiomyocyte mitosis. Activation of FGFR1 or FGFR2 has been demonstrated to have a positive effect on vascular endothelial proliferation. The first step of angiogenesis is ECM degradation. For instance, FGF1, FGF2, and FGF4 promote the expression of matrix metalloproteinases (MMPs) in endothelium cells. The second step in angiogenesis is endothelium migration. FGF1, FGF2, FGF8, and FGF10 were demonstrated to stimulate endothelium chemotaxis [67]. In clinical studies, FGF4 showed positive results in patients with stable angina following a series of AGENT-trials representing a single intracoronary infusion of adenovirus type 5 vector (Ad5FGF-4) [68]. In an ASPIRE trial, a single intracoronary infusion of Ad5FGF-4 using catheter-based administration was compared to standard care without a placebo group. It was planned to recruit 100 participants; however, the study was terminated apparently due to a low number of patients [69].

Another promising pro-angiogenic factor is HGF. It participates in mediation of angiogenesis by inducing endothelial cell proliferation, migration and survival. The HGF receptor c-Met is reported to be expressed on vascular endothelial cells. HGF/c-Met signaling induces endothelial cell proliferation through the MAPK/ERK and STAT3 pathways. In addition to the direct effect on stimulating endothelial cells to form tubular structures, HGF can recruit bone marrow endothelial progenitor cells to participate in angiogenesis in the ischemic area [70]. For cardiac tissue regeneration, HGF either in plasmid or adenoviral constructs has been tested but only in a small number of studies. Preclinical studies demonstrated the therapeutic effects of adenovirus carrying the HGF gene (Ad-HGF) in a minipig model of chronic myocardium ischemia in which an Ameroid constrictor was placed around the left circumflex branch of the coronary artery. The data showed that Ad-HGF significantly improves the heart function and blood supply in chronic myocardium ischemia models [71]. Furthermore, a catheter-based intramyocardial delivery (NavX) of Ad-HGF was proved to be safe and feasible for Ad-HGF delivery in pigs [70,72]. In a study by Rong and colleagues, skeletal myoblasts were transduced with Ad-HGF. Transplantation of HGF-engineered skeletal myoblasts results in reduced infarct size and collagen deposition, increased vessel density and improved cardiac function in a rat MI model [73]. The effect might be explained by the overexpression of HGF in skeletal myoblasts that confers resistance to ischemia in MI. Based on preclinical studies, the safe application of Ad-HGF was additionally confirmed by clinical trials. One such application is the use of two delivery approaches: an intramuscular myocardium direct injection while performing coronary artery bypass surgery (CABG) and a catheter-based intramyocardial injection guided by the NavX system. An open-label safety trial of Ad-HGF by a direct multipoint injection into the myocardium of patients, who suffer from coronary heart disease, showed that no subjective or objective adverse reactions were detected. Furthermore, there was evidence of revascularization in ischemic regions confirmed by instrumental examination and physical symptoms [71].

Additionally, some studies have reported the use of the stem cell factor (SCF), which is a ligand for the c-kit that is a receptor tyrosine kinase. SCF binding to c-kit leads to receptor dimerization and activation of multiple signaling pathways related to cell recruitment, differentiation, angiogenesis, and survival [74]. It has been shown that local overexpression of SCF post-MI induces the recruitment of c-kit+ cells at the infarct border area. Moreover, gene transfer of membrane-bound human SCF improved cardiac function in the model of ischemic cardiomyopathy in Yorkshire pigs [75].

The poor angiogenic nature of the adult heart prompted scientists to establish new therapeutic approaches for cardiac tissue regeneration. Thus, the effectiveness of gene-based therapy using pro-angiogenic factors may represent a promising therapeutic strategy to improve cardiac function. However, even though some clinical trials have shown promising results, further large-scale clinical trials need to be performed to clarify their efficacy and potential clinical application.

### 3.2. Gene Therapy for Scar Tissue Remodeling

Scar tissue forming after MI has a profound impact on the physiology of cardiac tissue. Since post-infarction scar is comprised of non-contractile fibrotic tissue, the scarred region of the heart can no longer contract and relax, leading to systolic and diastolic dysfunctions [76,77]. In addition, the fibrotic area becomes the source of various arrhythmias because it interferes with the conductive system of the heart [76,77]. Therefore, patients surviving MI are at a greater risk for morbidity and mortality due to cardiac dysfunction and arrhythmogenicity caused by the scar tissue.

Several gene therapy strategies have been successfully utilized for scar tissue remodeling (Figure 1). One of which is the direct reprogramming of cardiac fibroblasts into functional cardiomyocytes using a set of specific transcription factors. In vitro studies found that such a transformation required just three factors—such as Gata4, Mef2c and Tbx5, which were collectively designated as GMT [78]. Further studies identified that the use of combinations of 3–4 of the following factors—GATA4, MEF2C, TBX5, HAND2, MESP1, NKX2.5, and MYOCD—could be as efficient as or even better than the original GMT combination [79]. Cardiac reprogramming using this gene transfer was further accomplished in murine MI models using retroviral and lentiviral vectors. The in vivo reprogramming induced the formation of more mature cardiomyocytes compared to the same procedure in vitro. In addition, it led to the reduction of fibrosis and improved cardiac contractility of the injured heart. Despite the fact that early preclinical studies using cardiac reprogramming have achieved formation of new cardiomyocytes, the efficiency of this transformation was relatively low, frequently less than 30–40% [79]. Therefore, recent studies have attempted to combine cardiac reprogramming with other strategies to enhance cardiac repair [80]. In one of the studies, E-twenty-six Variant transcription factor 2 (ETV2) was introduced along with GMT genes into the genome of rat MI models using adenoviral and retroviral vectors, respectively [81]. ETV2 is crucial for the development of new blood vessels both during embryonic development and repair after injury [82]. Genetic therapy with a combination of ETV2 and GMT had positive effects on the recovery of the infarcted hearts. Namely, it induced the formation of new myocardium and blood vessels, reduced scar size and improved left ventricular function. Notably, the combinational gene transfer of GMT and ETV2 exhibited synergistic effects, i.e., the beneficial effects of the dual therapy were significantly more pronounced compared to individual gene transfers.

Direct cardiac reprogramming using conventional retroviral and lentiviral vectors possesses several limitations, such as low efficiency of fibroblast to cardiomyocyte transdifferentiation as well as potential insertional mutagenesis [83]. In order to address these obstacles, alternative gene transfer strategies for reprogramming of fibroblasts in the scar tissue have been considered. Isomi and colleagues have used Sendai virus as a vector to introduce GMT genes into cardiac fibroblasts of a mouse MI model [84]. The gene transfer induced the differentiation of fibroblasts into cardiomyocytes which in turn led to reduction of scar tissue and improved contractile function of the heart for 12 weeks. Importantly, this is the first study which observed long-term effects of the GMT-Sendai virus vector-based reprogramming. Another alternative gene carrier for direct cardiac reprogramming is nanoparticles. In a study by Chang and colleagues [83], cationic gold nanoparticles loaded with GMT transcription factor genes effectively induced transdifferentiation of mouse and human fibroblasts into cardiomyocytes in vitro, which was confirmed by their morphology and expression of cardiac-specific genes. Moreover, the direct injection of a gold nanoparticles-GMT construct into the infarct area of the murine heart generated more mature cardiomyocytes compared to in vitro experiments. In addition, the treatment resulted in a significant decrease in the scar dimensions two weeks after MI in mice. Importantly, it was shown in this study that the gold nanoparticles-carrier system did not cause integration of the delivered DNA into the host genome [83].

Besides direct cardiac reprogramming, gene therapy to attenuate fibrosis and scar tissue was also accomplished via ablation of genes regulating fibrosis. In a recent study, Adapala and colleagues [85] investigated the function of the transient receptor potential vanilloid 4 (TRPV4) mechanosensitive ion channel in cardiac remodeling after MI using TRPV4-knockout mice. The absence of TRPV4 was associated with a significant reduction in scar size and, remarkably, the presence of viable myocardium in the infarction area eight weeks after MI. Consequently, TRPV4-KO mice had a higher survival rate, and preserved systolic and diastolic function. The study also demonstrated that TRPV4 mediated differentiation of cardiac fibroblasts into myofibroblasts via a Rho/Rho kinase/MRTF-A pathway. This was evidenced by the observations that inhibition of TRPV4 caused reduced activity of Rho kinase as well as activation of TRPV4 with transforming growth factor-β1 (TGF-β1) or GSK (TRPV4 agonist) that led to the increased expression of MRTF-A. In another study, genetic ablation of Cullin-RING E3 ubiquitin ligase 7 (CRL7) was performed in order to attenuate fibrosis caused by pressure overload [86]. In particular, DNA recombination using Cre-recombinase transferred by AAV9 vector was utilized to obtain CRL7-knockout mice. Next, the mice were subjected to transverse aortic constriction, which is used to model chronic pressure overload and consequent fibrosis. CRL7 knock-out resulted in a 50% decrease in myocardial interstitial fibrosis and enhanced survival of cardiomyocytes. The observed beneficial effects of CRL7 genetic ablation were probably due to enhanced activation of a phosphatidylinositol 3-kinase (PI3K)/protein kinase B (Akt) signaling pathway, which regulates cell growth, proliferation, and apoptosis among other processes. In fact, the authors claim that the study is the first one that elucidated the role of CRL7 in PI3K/Akt regulation in cardiac tissue. Another signaling pathway that was found to be regulated by CRL7 was related to TGF-β1. Thus, the absence of CRL7 led to lower expression of TGF-β1 that confirms the pro-fibrotic role of CRL7. In another study, the role of low-density lipoprotein receptor-related protein 6 (LRP6) in cardiac repair after MI was investigated using miRNA-mediated cardiomyocyte-specific knock-down of the corresponding gene [87]. The depletion of LRP6 was found to be associated with a significantly smaller scar size and improved cardiac function in a mouse model of MI. Remarkably, it also caused enhanced proliferation of cardiomyocytes via the ING5/P21 pathway. It is important to note that the new cardiomyocytes originated from the existing cardiomyocytes rather than cardiac progenitor cells. This study is one of the first reports on the functions of LRP6 in cardiomyocyte proliferation in the adult heart. In summary, scar tissue remodeling using gene therapy can be accomplished by direct cardiac reprogramming as well as by ablation of fibrosis-related genes.

### 3.3. Gene Therapy to Regulate Reactive Oxygen Species

MI is one of the most common causes of death around the world. MI causes death of cardiomyocytes and irreversible damage to the heart tissue forming an infarcted zone, which is remodeled into fibrotic/scar tissue [88]. Mitochondrial damage is directly associated with cardiomyocyte death and myocardial dysfunction by inducing oxidative stress [89,90]. In addition, hypoxic stress resulting from MI leads to accumulation of reactive oxygen species (ROS), which causes apoptosis of cardiomyocytes and cardiac tissue injury. Most of the MI therapies aim to restore blood flow using CABG and percutaneous coronary intervention, which leads to an increase in the concentration of oxygen in ischemic cells [91]. This sharp increase in the amount of oxygen also induces the high production of ROS, causing cardiomyocyte injury. This process is called ischemia reperfusion injury (IRI) [92]. Moreover, regeneration of adult cardiomyocytes is limited and results in post-infarction cardiac dysfunction [91].

Most gene therapies aimed to eliminate post-infarction ROS are interested in the activation of antioxidant genes or inhibition of ROS producing genes. One of those studies investigated the effect of brahma-related gene 1 (BRG1), which regulates the chromatin structure and cardiac gene expression in Nuclear erythroid 2-related factor 2/Heme oxygenase 1 (Nrf2/HO-1) pathway [93]. Nrf2/HO-1 pathway is known to be a main regulator of cellular antioxidant responses. Translocation of Nrf2 to the nucleus activates the HO-1 and downstream oxidative stress pathway (HO1, glutathionetransferase p1 (GSTP1) or NAD (P)H:quinone oxidoreductase 1 (NQO1)) [94]. According to Liu and colleagues, overexpression of BRG1 gene by intramyocardial injection of adenoviral vectors induces the translocation of Nrf2 and attenuates the oxidative damage in cardiomyocytes. Moreover, the conducted studies demonstrated that Nrf2 expression, which was induced by BRG1, increased the activity of HO-1 promoters. On the other hand, Lenti-Brg1 shRNA injection, which inhibits the BRG1, showed adverse effects decreasing HO-1 expression [93]. Overall, BRG1 overexpression reduced the size of the infarct scar and improved the function of the cardiac tissue by decreasing oxidative damage and cell apoptosis. The HO-1 antioxidant molecule is also regulated by HIF-1α. HIF-1α overexpression with the help of a plasmid transfection encapsulated in 20-mer peptide-conjugated carboxymethylchitosan nanoparticle in MI mice upregulated the HO-1 activation and expression, which decreased ROS accumulation both in vitro and in vivo. Moreover, findings proved that downregulation of ROS-mediated oxidative stress inhibits the expression of BNIP3, which is responsible for cardiomyocyte apoptosis [95].

Another group of scientists investigated the effect of Zinc finger protein ZBTB20 in the treatment of post-infarction cardiac tissue. ZBTB20 was delivered with the help of AAV9 system [96]. Based on the results, overexpression of ZBTB20 in post-MI heart increased the superoxide dismutase (SOD) enzymatic activity, an important antioxidant that converts superoxide radicals to molecular oxygen, provided cellular defense and inhibited the activity of malondialdehyde (MDA) and NADPH oxidase [96,97].

Some of recent findings focus on the amelioration of mitochondrial dysfunction to eliminate ROS and to improve post-infarct cardiac function. Transverse aortic constriction (TAC) preconditioning of mice before the left coronary artery ligation induced MI showed a significant reduction in oxidative stress and decreased mitochondrial ROS production. TAC preconditioning can also be mimicked by the cardiac overexpression of SIRT3 in vivo with the help of AAV-SIRT3 transfection. Both TAC preconditioning and SIRT3 gene overexpression considerably increased the contractile function of heart and, in contrast, decreased the myocardial scar area and death of cardiomyocytes [98]. In addition, expression of deacetylated isocitrate dehydrogenase 2, a protein that regulates mitochondrial redox, reduced the production of mitochondrial ROS and alleviated post-MI injury [98,99].

Furthermore, there has been much research done recently to study the effects of miRNA, an important regulator in the development and pathophysiology of the cardiovascular system, on oxidative stress after MI [100]. Expression of miR-323-3p, Bax, Bcl-2, SOD1, and SOD2 genes and oxidative stress in cardiomyocytes (H9c2 cells) of miR-323-3p transfected mice were compared to mice without transfection and with miR-323-3p transfected and H_2_O_2_ treated group at seventh day after MI. Results indicated that miR-323-3p was downregulated in MI heart and overexpression of miR-323-3p decreased Bax and significantly increased Bcl-2, SOD1, and SOD2, consequently decreasing ROS production. Moreover, apoptosis of H9c2 cells decreased and cardiac function of mice improved considerably by targeting the TGF-β2/c-Jun N-terminal kinase (JNK) pathway [101]. Overexpression of miRNA-187 also attenuated the production of ROS by increasing the expression of SOD and reducing the intracellular MDA level, while inhibition of miRNA-187 had an inverse effect under the hypoxia/reoxygenation conditions of cardiomyocytes. This was achieved by the inhibition of DYRK2, a critical regulator of cell cycle and apoptosis, using miRNA-187, which consequently reversed the oxidative stress and apoptosis induced by cardiomyocyte hypoxia/reoxygenation conditions [102]. In addition, upregulation of miR-340-5p, which has an inhibitory effect on apoptosis, could suppress oxidative stress and apoptosis after hypoxia/reoxygenation of H9c2 cardiomyocytes by regulating the expression of Akt1 that upregulates the JNK and NF-κB signaling pathways, the main mediators of cell apoptosis [103]. Similarly, an aging-regulated long non-coding RNA (lncRNA) Sarrah demonstrated a protective effect against cardiomyocyte apoptosis by directly targeting nuclear factor erythroid 2-related factor 2 (NRF2) gene, which regulates the expression of antioxidant proteins that protect against oxidative damage. In an MI mouse model, overexpression of lncRNA Sarrah reduced cardiomyocyte apoptosis, while inducing endothelial cell proliferation and augmenting cardiac contractile function [104]. In contrast, other studies demonstrated that miRNA-124 is upregulated after MI under oxidative stress [105,106]. Application of antisense inhibitor oligodeoxyribonucleotides (AMO-124) alleviated the oxidative stress by neutralizing the miRNA-124, whose main target is STAT3, a key cellular survival factor that suppresses the apoptosis pathway [105]. Overall, gene therapy, specifically the application of various miRNAs, antioxidant gene activators and molecules, whose target is oxidative stress, is a very promising approach to eliminate ROS after MI and hypoxia/reoxygenation conditions and to improve cardiac function.

### 3.4. Gene Therapy to Reduce Apoptosis

Apoptosis has a significant impact on the regeneration of myocardial cells. Excessive apoptosis following hypoxia, oxidative stress and endoplasmic reticulum stress can contribute to the development of myocardial ischemia, IRI, cardiac remodeling, and atherosclerosis [107]. It also significantly increases the death of cardiomyocytes in ischemic heart disease (IHD) [108]. Therefore, targeting apoptotic agents and pathways can be an efficient strategy to promote cardiac tissue regeneration after CVDs. In the previous chapter, alleviation of oxidative stress using gene therapy to prevent apoptosis was discussed. In this section, other strategies to suppress apoptosis, namely, those that target molecular pathways involved in cellular death and survival will be covered.

Non-coding RNAs (ncRNAs) such as miRNAs, lncRNAs and circular RNAs (circRNAs), play an important role in the regulation of physiologic and pathologic signaling pathways in cardiomyocytes and can be used to regulate apoptosis in CVDs and improve cardiac tissue regeneration [109]. Yan and colleagues demonstrated that in vitro overexpression of miR-31a-5p, a leading member of the miRNA-31 family, can protect against apoptosis and increase myocardial cell survival through suppression of angiotensin II-induced apoptotic pathway and caspase-3 activity by targeting Tp53 [110]. Another miRNAs, miR378 *, demonstrated a cardioprotective effect by inhibiting endoplasmic reticulum stress-induced cell apoptosis via controlling expression of calcium-binding protein called calumenin in doxorubicin (Dox)-induced cardiomyopathic mouse hearts [111]. Moreover, another miRNA, miR-181c, was found to be suppressed in a mouse model of Dox-induced HF and its overexpression impeded cardiomyocyte apoptosis via PI3K/Akt pathway [112]. At the same time, it was reported that lncRNA UCA1 protects rat cardiomyocytes against hypoxia/reoxygenation induced apoptosis by inhibiting miR-143/MDM2/p53 signaling axis [113]. Moreover, adenovirus mediated expression of lncRNA GAS5 also decreases cardiomyocyte apoptosis through downregulation of transmembrane protein sema3a in an MI mouse model [114]. LncRNA FTX is also downregulated after IRI injury and enhanced expression of FTX attenuates cardiomyocyte apoptosis by targeting miR-29b-1-5p and Bcl2l2 [115]. Finally, a circRNA circ-Amotl1 demonstrated its ability to facilitate the cardio-protective nuclear translocation of pAkt by binding to both phosphoinositide dependent kinase-1 (PDK1) and Akt1. Injection of a circ-Amotl1 plasmid resulted in increased cardiomyocyte survival, decreased apoptosis and demonstrated a protective effect against Dox-induced cardiomyopathy in mice [116]. Zhu and colleagues also reported that AAV9 mediated cardiac overexpression of circRNA SNRK can target miR-103-3p by promoting cardiac repair via GSK3β/β-catenin pathway in rats with MI. Particularly, it reduced apoptosis, promoted cardiomyocyte proliferation, improved cardiac functions and increased angiogenesis [117]. Furthermore, Garikipati and colleagues demonstrated that AAV9 mediated delivery of circRNA CircFndc3b decreased cardiac apoptosis, enhanced neovascularization and left ventricle (LV) functions by interacting with the RNA binding protein fused in Sarcoma to regulate expression of VEGFs [118]. Therefore, these findings demonstrate that gene therapy with ncRNAs has great potential as a therapeutic strategy to protect against apoptosis in CVDs. However, further preclinical studies are needed to determine the anti-apoptotic effects of ncRNAs in large animal models.

Another strategy against cardiac apoptosis is to target genes encoding S100 family proteins. S100 proteins are highly acidic calcium-binding proteins involved in intracellular calcium homeostasis in different tissues and organs. Several members of this family are also expressed in cardiac tissue and their impairment can lead to the development of HF [119]. S100A1, a predominant member of the S100 protein family, is a crucial regulator of cardiac contractility. Due to its ability to enhance sarcoplasmic reticulum Ca^2+^ fluxes and increase SERCA2a enzyme activity, it can lead to the significant limitation of myocardial necrosis and development of HF [120]. A recent study conducted by Jungi and colleagues reported that S100A1 gene overexpression through the AAV9 vector can have cardioprotective effects in male Lewis rats after IRI. Particularly, S100A1 overexpressing hearts demonstrated improvement in LV functions, whereas the presence of necrotic markers, including troponin T (TnT), lactate dehydrogenase (LDH), and FoxO pro-apoptotic transcription factor were decreased [121]. Furthermore, a new liquid chromatography tandem mass spectrometry (LC-MS/MS)-based bioanalytical method was utilized for simultaneous quantitation of high homologous human and pig S100A1 proteins (only a single amino acid difference between the sequences). Therefore, the ability to accurately measure human S100A1 in pig hearts can enable future gene therapy studies in large animals [122]. Another member of the S100 family, S100A6, increases in the peri-infarct zone after MI in rats and protects cardiomyocytes from TNF-α-induced apoptosis by binding to p53 and decreasing its phosphorylation [123]. It was recently demonstrated that S100A6 gene delivery and its further cardiac overexpression attenuates cardiomyocyte apoptosis and reduces infarct size after myocardial IRI in both in vitro and in vivo models [124]. Thus, these results demonstrate that gene therapy mediated S100 protein expression can be an efficient method to attenuate cardiac apoptosis and protect cardiomyocytes against IRI injury.

Genetically modified stem cells can also be applied to protect against apoptosis by enhancing expression of various anti-apoptotic genes and factors. Cho and colleagues demonstrated that overexpression of lymphoid enhancer-binding factor-1 (LEF1) gene in human umbilical cord blood-derived mesenchymal stem cells (hUCB-MSCs) increased their cell proliferation and protected from hydrogen peroxide-induced apoptosis in in vitro experiments. Moreover, construction of hUCB-MSCs with overexpressed LEF1 using CRISPR/Cas9 system and further transplantation, demonstrated an enhanced cell survival rate and increased cardio-protective effects in an animal model of MI [125]. In another recent study, interleukin (IL)-10 gene was transfected into the genomic locus of amniotic mesenchymal stem cells (AMM) using transcription activator–like effector nucleases (TALEN). Further transplantation of IL-10 gene-edited AMM in an MI mouse model decreased the number of apoptotic cells and increased capillary density in an ischemic heart and as a result, increased LV functions and reduced infarct size [126]. At the same time, transplantation of bone marrow derived MSCs edited by CRISPR activation system to overexpress IL-10 also decreased apoptosis of cardiac cells, increased angiogenesis, and inhibited infiltration and production of proinflammatory factors in a diabetic MI mouse model [127]. Therefore, the therapeutic effects of genetically modified stem cells can be beneficial against apoptosis caused by cardiac ischemia. In summary, gene therapy with ncRNAs, S100 proteins or modified stem cells can be a potential and efficient strategy against cardiomyocyte death by targeting molecular pathways involved in apoptosis and cell survival in heart diseases.

## 4. Recent Ongoing Clinical Trials

Currently, several ongoing clinical trials are investigating the effects of various gene therapies on cardiac regeneration in CVDs. According to the ClinicalTrials.gov (accessed on 12 July 2021), there are a total of seven gene therapy trials, including six studies that are actively recruiting (EXACT, ReGenHeart, Korean trial, NAN-CS101) or planning to recruit (AFFIRM, CUPID-3) and one active clinical trial (EPICCURE).

The EXACT Trial is a phase I/II, first-in-human, multicenter, open-label, single arm dose escalation study recruiting 44 patients with refractory angina caused by CAD to evaluate the induction of therapeutic angiogenesis in ischemic myocardium by XC001 (AdVEGFXC1) [NCT04125732]. AdVEGFXC1 is an adenovirus type-5 vector expressing the hybrid VEGF with its three major isoforms (121, 165, and 189) [128]. In this trial, administration is at various doses by transthoracic epicardial procedure to 12 patients, followed by an increase in the maximum tolerated dose with 32 additional patients, and the main outcome measurements will be safety and side effect assessment.

ReGenHeart is a randomized, double-blinded, placebo-controlled multicenter phase II study enrolling patients with refractory angina pectoris to evaluate safety and efficacy of AdVEGF-D gene transfer [NCT03039751]. The trial is recruiting 180 participants in six different centers to whom revascularization cannot be performed. AdVEGF-D will be injected into myocardium through a catheter at 10 different sites and compared with a similar placebo treatment. The primary endpoint is to assess improvement in exercise tolerance and relief of angina symptoms at six months after injection.

EPICCURE is a randomized, placebo-controlled, double-blind, multicenter, phase II clinical trial testing the effect of VEGF-A165 mRNA loaded in biocompatible citrate-buffered saline (AZD8601) in patients with decreased LV function and undergoing CABG [NCT03370887] [129]. AZD8601 will be injected during the surgery as 30 epicardial injections in a 10-min extension of cardioplegia under the control of quantitative 15O-water positron emission tomography (PET) imaging. At the moment, 11 patients are enrolled in the study, and the primary endpoint is to investigate safety and tolerability of the gene therapy up to six months after the surgery. MRNA-based technology used in the EPICCURE trial is a novel revolutionizing approach for gene delivery that can be utilized for a variety of purposes such as protein replacement, gene editing, infectious diseases, cancer vaccines, and others [130]. There are multiple advantages of using an mRNA approach over conventional DNA-based strategies [131]. Firstly, unlike DNA, mRNA does not require entering the nucleus to be functional, making it more efficient. Secondly, mRNA-based strategy is much safer compared to DNA delivery with viral vectors since mRNA does not integrate into the host genome, eliminating the risk of insertional mutagenesis. Finally, as it has been proven by SARS-CoV-2 mRNA-based vaccines, mRNA production can be very rapid and capable of adapting to even high emergency conditions like pandemics. Indeed, two SARS-CoV-2 mRNA-based vaccines approved by the US Food and Drug Administration, Pfizer/BioNTech (BNT162b2), and Moderna (mRNA-1273), have been shown to be highly efficient in protecting against COVID-19 with 90–95% efficiency [132]. Moreover, a recent study published in *Nature* found that the two mRNA vaccines induced a persistent and robust germinal center response indicating that these vaccines produced strong and efficient humoral immune responses [133]. The benefits of mRNA-based technology have been utilized for cardiovascular gene therapy as well [134]. Specifically, mRNA therapeutics using genes for VEGF, IGF-1, and other proteins for heart regeneration were shown to be safe and effective in various animal models.

The AFFIRM study is a phase III clinical trial designed to determine the effect of intracoronary infusion of the human FGF-4 DNA sequence encoded in Ad5FGF-4 on ameliorating refractory angina symptoms and improving patient quality of life. This study will enroll 160 patients with refractory angina and the primary endpoint is the change of exercise tolerance test (ETT) duration over six months after intervention (NCT02928094). However, previous clinical trials with Ad5FGF-4 have demonstrated specific beneficial effects of this gene therapy only in females, while no difference was observed between placebo and treatment groups among male patients [68].

A phase II trial is ongoing in Korea to test the safety and efficacy of VM202RY, which is a DNA plasmid loaded with two isoforms of HGFs (723 and 728) (NCT03404024). A previous phase I trial demonstrated that VM202RY can improve cardiac perfusion and inhibit cardiac remodeling in patients with MI and angina [135]. Now they are recruiting 108 patients with acute MI for transendocardial injection of VM202RY through catheter to evaluate its safety, tolerability, and efficacy compared with placebo. Primary outcome measurements are to determine the maximum tolerated dose and assess LV ejection fraction after six months. Estimated study completion date was April, 2020, however, no results have yet been reported in English.

The CUPID-3 study is a randomized, double-blind, placebo-controlled phase I/II trial investigating the effect of SRD-001, an adeno-associated virus serotype 1 vector expressing the transgene for sarcoplasmic/endoplasmic reticulum Ca^2+^ ATPase 2a isoform (SERCA2a), on patients with cardiomyopathy and HF with reduced ejection fraction (NCT04703842). According to the authors, targeting SERCA2a enzyme expression by SRD-001 can correct defective intracellular Ca^2+^ hemostasis, subsequently improving cardiac contractility. In addition, SRD-001 can also correct the impaired endothelium-dependent vasodilatation, improve coronary blood flow and prevent further progression of HF. To determine this, 56 participants will be enrolled in the study until June 2021 and SRD-001 will be delivered through one-time intracoronary infusion. As a primary endpoint, change from baseline in symptomatic parameters (quality of life, HF severity), physical parameters, LV function/remodeling, and rate of recurrent and adverse events will be measured up to 12 months (NCT04703842).

NAN-CS101 is a phase I, prospective, multi-center, open-label, sequential dose escalation study to test the safety and efficacy of one dose intracoronary infusion of BNP116.sc-CMV.I1c in patients with HF (NYHA class III) (NCT04179643). BNP116.sc-CMV.I1c is an AAV vector with a chimeric AAV2/AAV8 capsid containing an active inhibitor of protein phosphatase-1 (I-1) transgene. Previous preclinical studies on large animals demonstrated that chimeric AAV vectors can selectively target cardiac muscle, while I-1 inhibits expression of protein phosphatase-1 and increases cardiac expression of SERCA2a enzyme leading to the improvement of cardiac functions [136,137]. In this small phase I study, 12 participants will be enrolled and injected intracoronary with different doses of BNP116.sc-CMV.I1. Primary endpoints will be the safety indicators measured by adverse events, mortality, and hospitalization up to 12 months after the procedure.

Lastly, another randomized, double-blind, placebo-controlled, phase II clinical trial has tested the effect of intracoronary delivery of adenovirus 5 encoding adenylyl cyclase 6 (Ad5.hAC6/RT100) in patients with HF. Adenylyl cyclase 6 (AC) is an enzyme that catalyzes the conversion of adenosine triphosphate to cyclic adenosine monophosphate and can have beneficial effects on cardiac myocytes and eventually improving LV function. Fifty-six subjects received intracoronary administration of Ad5.hAC6 or placebo and were followed up to 1 year. AC6 gene transfer safely increased LV function compared to a standard single-dose HF therapy and decreased admission rate in the treatment group [138]. A larger phase III trial (FLOURISH) with the same drug was recently registered in ClinicalTrials.gov (accessed on 12 July 2021); however, the current status of the study is withdrawn due to the reevaluation of “clinical development plans and strategy for RT-100” (NCT03360448). Therefore, despite its withdrawn status, a newly designed trial with Ad5.hAC6/RT100 can be conducted in the future. Table 1 summarizes the ongoing clinical trials.

Overall, most ongoing trials related to cardiac regeneration are focused on gene therapy targeting the expression of different growth factors and their isoforms to improve angiogenesis. This might be justified by the fact that extended angiogenesis effectively improves vascular and cardiac tissue regeneration leading to the enhancement of cardiac functions. However, there are still two gene therapy studies targeting the enzyme expression (CUPID-3 and NAN-CS101). Thus, this data demonstrates that gene therapy can be an efficient and safe approach to improve cardiac regeneration and further implementation of this strategy into clinical trials has the potential to develop into novel methods of treatment for CVDs.

## 5. Conclusions

The substantial health and economic burden of CVDs could be alleviated in the future with the advancement of novel therapeutic strategies that target pathogenetic mechanisms of the diseases. Gene therapy has been considered as a promising treatment option for a variety of CVDs. As it was reviewed in this paper, multiple preclinical studies have succeeded in reversing the outcomes of ischemic heart disease and MI by targeting angiogenesis, stimulating regeneration of cardiomyocytes, and alleviating oxidative stress and apoptosis. However, it will likely take many years for the translation of these beneficial effects from animal models to human patients. Many clinical trials of gene replacement therapy did not achieve efficiency, which might be associated with concentration of the replaced gene and its product at the target site and/or insufficient knowledge about pathogenetic mechanisms of CVDs. Moreover, safety considerations including potential off-target effects, tumorigenesis of certain viral vectors and arrhythmogenesis should be addressed before implementation of gene therapy in clinical practice. In addition, it is important to mention that individual use of the strategies described in this paper will most likely be insufficient in achieving complete heart regeneration after injury in humans. The main reason for this is that the pathways activated during cardiac injury are diverse and complex involving multiple cells and signaling cascades. Therefore, an efficient gene therapy for clinical use will likely combine several approaches described in this review—including but not limited to—strategies targeting fibrosis, oxidative stress, apoptosis, angiogenesis, and other processes.

## Figures and Tables

**Table 1:** Ongoing clinical trials in gene therapy for cardiac tissue regeneration in CVDs.

#	Study Title	Disease	Treatment Mechanism (Intervention)	Estimated Enrollment	Current Status and Phase	Trial Number
1	Epicardial Delivery of XC001 Gene Therapy for Refractory Angina Coronary Treatment (EXACT)	Coronary Artery Disease, Ischemia, Angina Refractory, Cardiovascular Diseases, Heart Diseases	Subjects in this study will receive one of four intramyocardial doses of XC001 that expresses human VEGF	44 participants	Recruiting, Phase I/II	NCT04125732
2	Adenovirus Vascular Endothelial Growth Factor D (AdvVEGF-D) Therapy for Treatment of Refractory Angina Pectoris (ReGenHeart)	Refractory Angina Pectoris, Coronary Artery Disease	A catheter mediated endocardial adenovirus-mediated VEGF-D (AdVEGF-D) regenerative gene transfer in patients with refractory angina to whom revascularization cannot be performed	180 participants	Recruiting, Phase II	NCT03039751
3	AZD8601 Study in CABG Patients (EPICCURE)	Heart Failure	Epicardial injections of VEGF-A165 mRNA formulated in biocompatible citrate-buffered saline (AZD8601) in a 10-min extension of cardioplegia during the CABG surgery under PET imaging	11 participants	Active, not recruiting, Phase II	NCT03370887
4	Ad5FGF-4 In Patients with Refractory Angina Due to Myocardial Ischemia (AFFIRM)	Angina, Stable	A single intracoronary infusion of an adenovirus serotype 5 virus expressing the gene for human FGF-4 (Ad5FGF-4)	160 participants	Not yet recruiting, Phase III	NCT02928094
5	Safety and Efficacy Study of Gene Therapy for Acute Myocardial Infarction	Ischemic Heart Disease, Acute Myocardial Infarction	VM202RY injected via transendocardial route using C-Cathez^®^ catheter in subjects with acute MI	108 participants	Recruiting, Phase II	NCT03404024
6	Calcium Up-Regulation by Percutaneous Administration of Gene Therapy in Cardiac Disease (CUPID-3)	Congestive Heart Failure, Systolic Heart Failure	A single intracoronary infusion of adeno-associated virus serotype 1 vector expressing the transgene for sarco(endo)plasmic reticulum Ca^2+^ ATPase 2a isoform (SRD-001)	56 participants	Not yet recruiting, Phase I/II	NCT04703842
7	NAN-101 in Patients with Class III Heart Failure (NAN-CS101)	Heart Failure, Heart Disease, Ischemic Cardiovascular Diseases, Heart Arrhythmia	A single intracoronary infusion of chimeric adeno-associated virus serotype 2/8 with an active I-1 transgene	12 participants	Recruiting, Phase I	NCT04179643
